# Post-traumatic stress disorders among Syrian refugees residing in non-camp settings in Jordan

**DOI:** 10.15537/smj.2023.44.1.20220701

**Published:** 2023-01

**Authors:** Iman A. Basheti, Shahnaz Mohammed Ayasrah, Rajaa Ali Al-Qudah

**Affiliations:** *From the Department of Clinical Pharmacy and Therapeutics (Basheti, Al-Qudah), Faculty of Pharmacy, Applied Science Private University, Amman; from the Department of Applied Science/Nursing (Ayasrah), Al-Balqa Applied University, Al-Salt, Jordan; from the Faculty of Medicine and Health (Basheti), Faculty of Pharmacy, The University of Sydney, Sydney, Australia; and from the Division of Pharmacy and Optometry (Al-Qudah), Faculty of Biology, Medicine and Health, The University of Manchester, Manchester, United Kingdom.*

**Keywords:** post-traumatic stress disorder, Syrian refugee, Jordan

## Abstract

**Objectives:**

To measure the prevalence and severity of post-traumatic stress disorders (PTSD) among Syrian refugees and explore its association with various factors.

**Methods:**

A cross-sectional study was carried out among a convenience snowball sample of Syrian refugees residing in non-camp settings in Jordan in 2019. A 4-part self-administered structured questionnaire was used to collect data. Part one included socio-demographic data, part 2 included an Arabic version of Harvard Trauma Questionnaire (HTQ) (part I: trauma event and part IV: trauma symptoms), part 3 was related to participants’ physical symptoms, and part 4 to participants’ satisfaction with the healthcare they received.

**Results:**

Study participants (n=279; mean age 32 years (SD=10.45), 52% were males) reported high prevalence of traumatic symptoms (86.2%); of these, 68.5% were considered symptomatic for PTSD (HTQ-16 sub-scale or the entire symptom scale HTQ-45 mean item score of >2.5), regardless of the type of trauma. Those who were middle-aged, a female, unemployed, sexually abused or raped, had a family member who died in the conflict, witnessed catastrophic events like burning, or razing of residential areas, and have received the body of a family member while being prohibited from expressing grief and doing funeral rites, were more likely to be considered as a case of PTSD.

**Conclusion:**

Majority of the refugees residing in non-camp settings in Jordan suffer from PTSD. Refugees have low satisfaction with the healthcare services provided.

**S**yria’s civil conflict, which began in March 2011, continues to be the source of the world’s highest number of forcibly displaced people.^1^ As of the end of 2017, 12.6 million Syrians had been forcibly displaced, with 6.3 million refugees, 146,700 asylum seekers, and 6.2 million internally displaced persons.^2^ The Syrian crisis was declared “the worst humanitarian crisis of the twenty-first century” by the United Nations.^3^ According to the most recent figures of the United Nations High Commissioner for Refugees, there were 654,266 registered refugees in Jordan as of November 2019, with 192,721 (29.5%) living in Amman, the capital.^3^

It has been well established that being exposed to war is usually associated with a lower quality of life among refugees.^4-6^ The effect of war on refugees’ lives is not temporary in most cases but persists over many years.^7,8^ Unsurprisingly, post-traumatic stress disorder (PTSD) experienced by refugees has been linked to poor self-reported quality of life.^9-12^ It is widely acknowledged to be the most prevalent consequence of violence among refugees.^13^ In order to be diagnosed with PTSD, a person must experience at least one month of recurrent, painful exposure to a traumatic experience, as well as emotional distress or hyper arousal.^13^

As a result of war and PTSD, lack of employment, poor daily activities, and inadequate living circumstances have been reported among refugees.^14^ A study by Matanov et al^15^ (2013) involving 854 refugees assessed the quality of life to explore the impact of war events, PTSD symptoms, and post-war environment on it in 5 countries of Balkan, and the results showed dissatisfaction among refugees with employment and financial situation. Unemployment and low contact with friends were also associated with a lower quality of life due to the post-war environment effect.^15^ In countries with a high influx of refugees, such as Sweden, the quality of life of refugees was rated lower than population norms and correlated negatively with mental health outcomes; high levels of psychological distress were also found.^16^ Quality of life was significantly lower for refugees who were 3 times more likely to have poor mental health, particularly PTSD, than for non-refugees.^5,10^

Basheti et al^1^ carried out a pharmacist-led study in Jordan in 2019 to assess the prevalence of PTSD among Syrian refugees living in Amman. The prevalence of PTSD was found to be 38.7% among the study participants. Poor living conditions that exacerbate respiratory illnesses and a lack of access to transportation to healthcare facilities within the camp were among the health-related issues for camp residents, according to a study carried out by Al-Rousan et al^17^ (2018) determined the health needs and priorities of Syrian refugees living in camps and other settings in Jordan. However, those who lived in non-camp environments, such as urban areas, expressed health problem concerns on limited access to secondary and tertiary care due to high costs to get to these places, high prevalence of chronic conditions like cardiovascular disease, diabetes, and hypertension since the displacement, as well as mental illnesses like PTSD and depression, which are rising in young adults. Many of the refugees commended for mental health treatments and highlighted money worries as their main source of stress.^17^ As refugees started moving out of the camps to reside in Jordan’s cities and towns, the demands on the country’s basic infrastructure, services, and resources increased. Communities in Jordan have been severely impacted by the refugee inflow, being more susceptible to communicable diseases, having less access to health care, and experiencing rising rates of morbidity, environmental difficulties, and social challenges.^18^ However, such refugees may experience poor health and have trouble obtaining medical care due to their situations. Additional elements that worsen their health quality include a lack of health insurance, the cessation of free access to health services, health care costs as well as cultural disparities.^19,20^ Assessing the rising health needs of Syrian refugees, particularly those residing in non-camp settings has been recommended by previous literature;^1,18,20^

This study aimed to measure the prevalence and severity of PTSD among Syrian refugees settled in non-camp settings in Jordan, explore its association with various sociodemographic variables and the participants’ characteristics, determine its predictors, assess the types of trauma that they have experienced, assess the physical symptoms associated with these traumas (symptoms that people who experience catastrophic accidents sometimes feel in their lives), assess how these symptoms affect their social life, and their satisfaction with the medical care provided for them.

## Methods

This study used a cross-sectional design. A convenience-based snowball sample of Syrian refugees residing in non-camp settings in Jordan was recruited for this study in 2019. Participants were included if they were aged 18 years and older, had Syrian nationality, were officially registered as refugees, could read and write in Arabic, and were willing to participate with written informed consent. Participants who were treated with psychotropic medicines, had severe hearing or visual impairments, or had been diagnosed with a mental disease or substantial psychiatric illness (such as dementia or depression) were all excluded from the study. Syrian refugees residing in selected areas in Jordanian cities that are highly populated with Syrian residents were approached using the snowball sampling method when it was most convenient for them.

The questionnaire and methodology for this study were approved by the Jordanian Ministry of Health, and all procedures were approved by the Institutional Review Board of the Applied Science Private University. Data were collected between June and December 2019. After full disclosure, the study participants were requested to sign an informed consent form and completed a self-administered written questionnaire. A trained psychotherapist with more than 5 years of experience in working with refugees attended the visits to the participants to clarify any ambiguous item. Most participants were accessed in their homes or work places, upon their request. To have a variety in participants experiences, only one participant, who achieved the inclusion criteria and willing to participate, was recruited from each family. Based on an initial analysis of the completed questionnaires, participants with severe symptoms, needing medical help were referred to psychotherapist or psychologist clinics based on appointments made by the psychotherapist who attended the visit, for further assessment. It was explained to the study participants that the information they provided would help them receive better care and would not have an impact on their lives or treatment plans, and that if any of the questions made them feel uncomfortable or embarrassed, they could choose not to answer, or, if they did, that their responses would be kept confidential. Self-administered surveys were filled out anonymously and de-identified by assigning each participant a code in order to maintain confidentiality. The study was carried out according to the Helsinki Declaration.

A self-administered structured questionnaire comprising 4 sections was used for data collection. Section one included questions related to sociodemographic and personal aspects (such as age, gender, employment, and marital status). Section 2 included 2 components of the Arabic version of the HTQ; part I: trauma event, and part IV: trauma symptoms,^21^ which was developed from the Diagnostic and Statistical Manual of Mental Disorders Text Revision (DSM-IV TR) published by the American Psychology Association (APA, 1987, 1994).^22^ The part I (trauma event) consisted of 42 questions that describe various stressors encountered by refugees, such as torture, rape, killing, and shortage of food or water, to explore the type of painful or horrific trauma events that evoked significant distress symptoms. Participants were asked if they had ever experienced, witnessed, or encountered an occurrence that involved real or threatened death, significant harm, or a threat to one’s own or another person’s physical integrity. The participants responded with 2 reaction options (yes or no). The part IV (trauma symptom) included 45 questions exploring the symptoms associated with the trauma felt by refugees. Part IV comprises of 2 parts: participants’ PTSD symptoms (16 items) and their self-perception of functioning (SPFS) (29 items), which includes questions regarding how trauma affects people’s perceptions of their capacity to operate in daily life. Separate PTSD symptoms and SPFS scores may also be computed in addition to the Part IV overall score (45 items).^22^ The answers to this part were rated on a 4-point Likert scale ranging from 1 (not at all) to 4 (extremely). Shoeb et al^23^ validated the Arabic version of the questionnaire among Iraqi refugees in the United States in 2007. The prevalence and severity of trauma symptoms were assessed using the mean item scores for the first HTQ-16 and the entire symptom scale (HTQ-45). A standard cutoff score of 2.5, as reported by Mollica et al^24^ (2004) was adopted to indicate probable PTSD.

The third section of the study questionnaire consisted of 15 questions, each question assesses a physical symptom (such as stomachache, backache, dysmenorrhea [for the female participants]) without identifying specific traumatic experiences, across the previous 4 weeks, using a 3-point scale ranging from “not bothered at all” to “bothered a lot.” These symptoms were selected based on previous literature.^25^ An additional question was added to this section to assess the degree to which psychological problems and physical symptoms interfered with social activities. Participants rated their responses using a 5-point Likert-type scale, ranging from 0 (never) to 5 (all the time). The fourth section included 13 questions that assessed refugees’ satisfaction with the lifestyle, medical care, and child healthcare that they received following their settlement in Jordan. Participants’ responses were rated on a 5-point Likert-type scale ranging from 0 “bad” to 5 “excellent”. To calculate the sample size, a 2-tailed independent samples t-test with a medium effect size of 0.40, a significant level of 0.05, and a statistical power of 0.80 was utilized. A total sample of 200 participants were needed to achieve a statistical power of 0.80 with a medium effect size using G*power. Another 30 additional participants have been included to account for attrition.

### Statistical analysis

Descriptive statistics were performed to describe sociodemographic and personal characteristics (such as the prevalence of PTSD, the types of trauma and the physical symptoms associated with these types, how these symptoms affect the refugees’ daily activities, and the level of satisfaction among refugees regarding the medical care they and their family have received). Categorical data are presented as number of cases (n) and frequencies (%). Continuous variables are expressed as mean ± standard deviation (SD). Statistical comparisons between various groups were conducted using Pearson’s Chi-square and Fisher’s exact tests if the assumption of (χ^2^) test was violated for categorical variables, and Student’s t-test or the Mann-Whitney U test for continuous variables, and *p*≤0.05 were considered significant. To determine whether there is a relationship between the study variables and being classified as a case of PTSD (mean score >2.5 on the HTQ-16’s sub-items or overall scale (HTQ-45), the Chi-square test was employed. A multivariate-adjusted logistic regression was used to examine the influence of the variables that were associated with the outcome variable on the likelihood that participants would be considered as a case of PTSD. The last category for each independent variable with more than 2 categories, such as educational level, was designated as the reference group when it was added to the model as a categorical covariate. The reference categories were chosen from other variables with 2 categories in accordance with the Statistical Package for the Social Sciences (IBMCorp, Armonk, NY, USA) version 21 was used to perform statistical analyses.

## Results

This study included a total of 279 Syrian refugees. Participants’ ages ranged from 18 to 62 with a mean age of 32 years (SD=10.45), and 52% were male. All participants belonged to the Muslim-Sunni religion. The number of family members, including the participant, ranged from 2 to 15 members, with a mean of 5.8 (SD=2.79). More than half of the participants were married (61.6%); however, 73% of the participants reported being unemployed at the time of data collection. On average, the participants spent 22.3 months in Syria during the conflict and 16.1 months in Jordan at the time of data collection. Nearly 56% of the study participants had at least one family member who died during the war; nevertheless, if peace restores there, 80% of them would return to their country. Regardless of the type of trauma, almost 86.2% of the participants had experienced trauma. Table 1 presents the sociodemographic variables of the participants based on being a case of PTSD.

**Table 1:** - Socio-demographics for the study participants based on being a case of PTSD or not* (N=279).

Characteristics	Non-case of PTSD n (%)	A case of PTSD n (%)	Total n (%)	χ2 *P*-value
***Age group (years)***				
Young (18-30)	47 (31.8)	135 (68.2)	182 (56.2)	χ2=9.19 *p*=0.01
Middle-aged (31-45)	36 (42.3)	45 (57.7)	45 (34.8)	
Old-aged (45)	6 (48.0)	10 (52.0)	10 (9.0)	
*Gender*				
Males	61 (41.8)	85 (58.2)	146 (52.3)	χ2=13.8 *p*<0.001
Females	28 (31.6)	105 (68.4)	133 (47.7)	
***Educational level***				
Illiterate	1(33.3)	2(66.7)	3 (1.1)	χ2=0.68 *p*=0.878
Primary	45 (34.1)	87 (65.9)	132 (47.3)	
Secondary	26 (30.9)	58 (69.0)	84 (30.1)	
University	17 (28.3)	43 (71.7)	60 (21.5)	
***Marital status***				
Single	26 (27.7)	68 (72.3)	94 (33.7)	χ2=2.84 *p*=0.42
Married	58 (33.5)	115 (66.5)	173 (62.0)	
Divorced	3 (60.0)	2 (40.0)	5 (1.80)	
Widowed	2 (28.6)	5 (71.4)	7 (2.50)	
***Employment status***				
Unemployed	57 (28.0)	147 (72.0)	204 (73.0)	χ2= 5.47 *p*=0.019
Employed	32 (42.7)	43 (57.3)	75 (27.0)	
***Having a family member died in the conflict***				
No	64 (51.6)	60 (48.4)	124 (44.4)	χ2= 39.9
Yes	25 (16.1)	130 (83.9)	155 (55.6)	*p*<0.001
***If peace restore, you would go back to your country***				
No	8 (14.0)	49 (86.0)	57 (20.4)	χ2= 10.5
Yes	81 (36.5)	141 (63.5)	222 (79.6)	*p*=0.001

Values are presented as number and percentage (%). *Mean score >2.5 on the Harvard Trauma Questionnaire (HTQ)-16’s sub-items or entire scale (HTQ-45) indicated a case of post-traumatic stress disorders (PTSD), χ2: Chi-square, p: significant level

Being symptomatic of PSTD (a case: mean score >2.5 on the HTQ-16 or the entire symptom scale) was seen in 68.5% of the study participants, regardless of the type of trauma they experienced. The mean score for the HTQ-16 was 2.5 (SD=0.62), ranging from 1.0 to 4.0, while the mean for the entire symptom scale (HTQ-45) was 2.43 (SD=0.54), ranging from 1.1 to 3.43. The most common type of trauma reported by the participants was expulsion from their country based on ancestral origin, religion, or sector (92%); of these, 68% were classified as PTSD cases. Overall, the study participants reported a total number of 5014 traumatic events (median=120.5, inetquartile range=154.25). The minimum number of trauma events was 8 and the maximum was 258 events; however, one participant may have reported more than one type of trauma. The highest percentage of being a case of PTSD among those who experienced any traumatic events was seen among those who experienced being confined to their home because of chaos and violence outside (70%). All participants who reported a catastrophic experience of being kidnapped (n=9) or sexually abused or raped (n=26) were classified as PTSD cases. In general, 16 of these traumatic events significantly related with being a case of PTSD. Examples of these catastrophic experiences include “witnessed shelling, burning, razing of residential areas or marshlands”, “witnessed mass execution of civilians”, “receiving the body of a family member such as a child or a spouse and being prohibited from mourning them and performing burial rites”, “the disappearance of a family member such as a child or a spouse as well as the disappearance of a friend”, “being sexually abused or raped, and sexual coercion” (χ^2^=32.0, χ^2^=17.4, χ^2^=15.2, χ^2^=13.4, χ^2^=12.72, *p*<0.001, sequentially). Table 2 shows the different types of traumatic events (n=40) reported by study participants based on being a case of PTSD or not.

**Table 2:** - Type of traumatic events reported by the study participants based on being a case of post-traumatic stress disorders (PTSD) or not* (N=279).

Type of traumatic event	Non-case of PTSD n (%)	A case of PTSD n (%)	Total n (%)	χ2 *P*-value
***“Oppressed because of ethnicity, religion, or sect”***				
No	52 (31.3)	114 (68.7)	166 (59.5)	χ2=0.010
Yes	36 (31.9)	77 (68.1)	113 (40.5)	*p*=0.921
***“Present while someone searched for people or things in your home”***				
No	8 (26.7)	22 (73.3)	30 (10.8)	χ2=0.370
Yes	80 (32.1)	169(67.9)	249 (89.2)	*p*=0.543
***“Searched”***				
No	33 (28.0)	85 (72.0)	118 (42.3)	χ2=1.21
Yes	55 (34.2)	106 (65.8)	161 (57.7)	*p*=0.271
***“Property looted or confiscated or destroyed”***				
No	21 (30.9)	50 (69.0)	71 (30.1)	χ2=0.170
Yes	67 (28.3)	141 (71.7)	208 (21.5)	*p*=0.680
***“Forced to leave your hometown and settle in a different part of the country with minimal services”***				
No	12 (27.3)	32 (72.7)	44 (15.8)	χ2=.441
Yes	76 (32.3)	159 (67.7)	235 84.2)	*p*=0.507
***“Imprisoned”***				
No	77 (33.3)	154 (66.6)	231 (82.8)	χ2=1.99
Yes	11 (22.9)	37 (77.1)	48 (17.2)	*p*=0.158
***“Suffered ill health without access to medical care or medicine”***				
No	33 (31.7)	71 (68.3)	104 (37.3)	χ2=0.003
Yes	55 (31.4)	120 (68.6)	175 (62.7)	*p*=0.958
***“Suffered from lack of food and cleaned water”***				
No	33 (30.3)	76 (69.7)	109 (39.1)	χ2=0.133
Yes	55 (32.4)	115 (67.6)	170 (60.9)	*p*=0.716
***“Forced to flee your country”***				
No	39 (14.0)	76 (86.0)	115 (20.4)	χ2=0.510
Yes	49 (36.5)	115 (63.5)	164 (79.6)	*p*=0.475
***“Expelled from your country based on ancestral origin, religion, or sect”***				
No	6 (24.0)	19 (76.0)	25 (9.0)	χ2=0.723
Yes	82 (32.3)	172 (67.7)	254 (91.0)	p=0.395
***“Lack shelter”***				
No	19 (14.0)	34 (86.0)	53 (20.4)	χ2=0.562
Yes	69 (36.5)	157 (63.5)	226 (79.6)	*p*=0.453
***“Witnessed the desecration or destruction of religious shrines or places of religious instruction”***				
No	9 (23.1)	30 (76.9)	39 (14.0)	χ2=1.50
Yes	79 (32.9)	161 (67.1)	240 (86.0)	*p*=0.220
***“Witnessed the arrest, torture, or execution of religious leaders or important members of tribe”***				
No	24 (39.3)	37 (60.7)	61 (21.9)	χ2= 2.20
Yes	64 (29.4)	154 (70.6)	218 (78.1)	*p*=0.138
***“Witnessed mass execution civilians”***				
No	70 (40.7)	102 (59.3)	172 (61.6)	χ2=17.4
Yes	18 (16.8)	89 (83.2)	107 (38.4)	*p*<0.001
***“Witnessed shelling, burning, or razing of residential areas or marshlands”***				
No	28 (70.0)	12 (30.0)	40 (14.3)	χ2= 32.0
Yes	60 (25.1)	179 (74.9)	239 (85.7)	*p*<0.001
***“Witnessed chemical attacks on residential areas or marshlands”***				
No	79 (35.7)	142 (64.3)	221 (79.2)	χ2=8.71
Yes	9 (15.5)	49 (84.5)	58 (20.8)	*p*<0.001

Type of traumatic event	Non-case of PTSD n (%)	A case of PTSD n (%)	Total n (%)	χ2 *P*-value
***“Exposed to combat situation (explosions, artillery fire, shelling) or landmine”***				
No	5 (21.7)	18 (78.3)	23 (08.2)	χ2=1.12
Yes	83 (32.4)	173 (67.6)	256 (91.8)	*p*=0.291
***“Serious physical injury from combat situation or landmine”***				
No	82 (32.9)	167 (67.1)	249 (89.2)	χ2=2.10
Yes	6 (20.0)	24 (80.0)	30 (10.8)	*p*=0.150
***“Used as a human shield”***				
No	76 (32.8)	156 (67.2)	232 (83.2)	χ2=. 950
Yes	12 (25.5)	35 (74.5)	47 (16.8)	*p*=0.331
***“Serious physical injury of family member or friend from combat situation or landmine”***				
No	44 (43.6)	57 (56.4)	101 (36.2)	χ2=10.60
Yes	44 (24.7)	134 (75.3)	178 (63.8)	*p*=0.001
***“Witnessed rotting corpses”***				
No	62 (38.3)	100 (61.7)	162 (58.1)	χ2=8.10
Yes	26 (22.2)	91 (77.8)	117 (41.9)	*p*=0.004
***“Confined to home because of chaos and violence outside”***				
No	10 (47.6)	11 (52.4)	21 (07.5)	χ2=2.72
Yes	78 (30.2)	180 (69.8)	258 (92.5)	*p*=0.099
***“Witnessed someone being physically harmed (beating, knifing, and so on)”***				
No	35 (39.3)	54 (60.7)	89 (31.9)	χ2=3.67
Yes	53 (27.9)	137 (72.1)	190 (68.1)	*p*=0.055
***“Witnessed sexual abuse or rape”***				
No	78 (34.5)	148 (65.5)	226 (81.0)	χ2=4.87
Yes	10 (18.9)	43 (81.1)	53 (19.0)	*p*=0.027
***“Witnessed torture”***				
No	49 (40.2)	73 (59.8)	122 (43.7)	χ2 =7.47
Yes	39 (24.8)	118 (75.2)	157 (56.3)	*p*=0.006
***“Witnessed murder”***				
No	55 (39.3)	85 (60.7)	140 (50.2)	χ2=7.81
Yes	33 (23.7)	106 (76.3)	139 (49.8)	*p*=0.005
***“Forced to inform on someone placing them at risk of injury or death”***				
No	84 (31.8)	180 (68.2)	264 (94.6)	χ2=0.174
Yes	4 (26.7)	11 (73.3)	15 (05.4)	*p*=0.676
***“Forced to destroy someone’s property”***				
No	86 (32.2)	181 (67.8)	267 (95.7)	χ2=0.961
Yes	2 (16.7)	10 (83.3)	12 (04.3)	*p*=0.327
***“Forced to physically harm someone (beating, knifing, and so on)”***				
No	87 (32.1)	184 (67.9)	271 (97.1)	χ2=1.38
Yes	1 (12.5)	7 (87.5)	8 (02.9)	*p*=0.240
***“Murder or violent death of family member (child, spouse, and so on)”***				
No	49 (39.2)	76 (60.8)	125 (44.8)	χ2=6.15
Yes	39 (25.3)	115 (74.6)	154 (55.2)	*p*=0.013
***“Received the body of a family member (child, spouse, and so on) and prohibited from mourning them and performing burial rites”***				
No	77 (38.3)	124 (61.7)	201 (72.0)	χ2=15.2
Yes	11 (14.1)	67 (85.9)	78 (28.0)	*p*<0.001
***“Disappearance of a family member (child, spouse, and so on) or disappearance of a friend”***				
No	63 (40.6)	92 (59.4)	155 (55.6)	χ2 =13.4
Yes	25 (20.2)	99 (79.8)	124 (44.4)	*p*<0.001

Type of traumatic event	Non-case of PTSD n (%)	A case of PTSD n (%)	Total n (%)	χ2 *P*-values
***“Family member (child, spouse, and so on) or friend kidnapped”***				
No	76 (39.3)	135 (60.7)	211 (21.9)	χ2 =10.8
Yes	12 (29.4)	54 (70.6)	66 (78.1)	*p*=0.001
***“Family member (child, spouse, and so on) or friend taken as a hostage”***				
No	76 (36.0)	135 (64.0)	211 (75.6)	χ2 =7.38
Yes	12 (18.2)	54 (81.8)	66 (78.1)	*p*=0.007
***Someone informed on you placing you at risk of injury or death***				
No	80 (33.5)	159 (66.5)	239 (85.7)	χ2 =2.65
Yes	8 (20.0)	32 (80.0)	40 (14.3)	*p*=0.103
***Physically harmed (beaten, knifed, and so on)***				
No	69 (31.1)	153 (68.9)	222 (79.6)	χ2=0.093 *p*=0.760
Yes	19 (33.3)	38 (66.7)	57 (20.4)	
***Kidnapped***				
No	88 (32.6)	182 (67.4)	270 (96.8)	χ2= 4.29 *p*=0.038
Yes	0 (0)	9 (100)	9 (03.2)	
***Taken as a hostage***				
No	83 (31.4)	181 (68.6)	264 (94.6)	χ2=0.02 *p*=0.878
Yes	5 (33.3)	10 (66.7)	15 (05.4)	
***Sexually abused or raped (such as forced sexual activity)***				
No	88 (39.3)	165 (60.7)	253 (90.7)	χ2=12.72
Yes	0 (0)	26 (100)	26 (09.3)	*p*<0.001
***Tortured (such as while in captivity you received deliberate and systematic infliction of physical and/or mental suffering)***				
No	79 (35.1)	146 (64.9)	225 (80.6)	χ2= 6.86 *p*=0.009
Yes	9 (16.7)	45(83.3)	54 (19.4)	

Values are presented as number and percentage (%). *Mean score >2.5 on the Harvard Trauma Questionnaire (HTQ)-16’s sub-items or entire scale (HTQ-45) indicated a case of PTSD, χ2: Chi-square, *p*: significant level

Over the last 4 weeks and regardless of the type of trauma, 79% of the participants reported experiencing some level of a bothered headache; of those, 36% reported experiencing a lot of headaches. Almost 34% of the participants reported experiencing a lot of pain in their arms, legs, or joints, feeling tired, or having low energy and sleep disturbances; of these, 85% and 90%, in the order given, were classified as cases of PTSD. Among female participants, 28% reported experiencing a lot of menstrual cramps or other period-related problems; of them, 92% were classified as cases of PTSD. Table 3 shows the rest of the physical symptoms exhibited by the study participants, based on the presence or absence of PTSD.

**Table 3:** - Physical symptoms experienced by the study participants based on being a case of PTSD or not* (N= 279).

Characteristics	Non-case of PTSD n (%)	A case of PTSD n (%)	Total n (%)	χ2 *P*-value
***Stomach pain***				
Not bothered at all	44 (41.5)	62 (58.5)	106 (38.0)	χ2=9.1 *p*=0.011
Bothered a little	29 (28.7)	72 (71.3)	101 (36.0)	
Bothered a lot	15 (20.8)	57 (79.2)	72 (26.0)	
***Back pain***				
Not bothered at all	34 (39.1)	53 (60.9)	87 (31.2)	χ2=7.99 *p*=0.018
Bothered a little	37 (34.6)	70 (65.4)	107 (38.3)	
Bothered a lot	17 (20.0)	68 (80.0)	85 (30.5)	
***Pain in your arms, legs, or joints (knees, hips, and so on)***				
Not bothered at all	45(46.4)	52(53.6)	97 (34.8)	χ2=23.0 *p*<0.001
Bothered a little	29 (33.7)	57 (66.3)	86 (30.8)	
Bothered a lot	14 (14.6)	82 (85.4)	96 (34.4)	
***Menstrual cramps or other problems with periods (female, n=133)***				
Not bothered at all	15 (28.3)	38 (71.7)	53 (39.9)	χ2=5.53 *p*=0.063
Bothered a little	10 (23.3)	33 (76.7)	43 (32.3)	
Bothered a lot	3 (08.1)	34 (91.9)	37 (27.8)	
***Headaches***				
Not bothered at all	30 (50.0)	30 (50.0)	60 (21.5)	χ2=25.9 *p*<0.001
Bothered a little	44 (37.3)	74 (62.7)	118 (42.3)	
Bothered a lot	14 (13.9)	87 (86.1)	101 (36.2)	
***Chest pain***				
Not bothered at all	27 (18.4)	90 (61.2)	147 (52.7)	χ2= 7.54 *p*=0.023
Bothered a little	20 (23.8)	64 (76.2)	84 (30.1)	
Bothered a lot	11 (22.9)	37 (77.1)	48 (17.2)	
***Dizziness***				
Not bothered at all	54 (42.5)	73 (57.5)	127 (45.5)	χ2= 17.3 *p*<0.001
Bothered a little	24 (29.6)	57 (70.4)	81 (29.1)	
Bothered a lot	10 (14.1)	61 (85.9)	71 (25.4)	
***Fainting spells***				
Not bothered at all	68 (32.1)	144 (67.9)	212 (76.0)	χ2=0.255 *p*=0.880
Bothered a little	14 (28.6)	35 (71.4)	49 (17.6)	
Bothered a lot	6 (33.3)	12 (66.7)	18 (6.4)	
***Feeling your heart pound or race***				
Not bothered at all	53 (40.2)	79 (59.8)	132 (47.3)	χ2=9.23 *p*=0.003
Bothered a little	27 (25.7)	78 (74.3)	105 (37.6)	
Bothered a lot	8 (19.0)	34 (81.0)	42 (15.1)	
***Shortness of breath***				
Not bothered at all	47 (42.7)	63 (57.3)	110 (39.4)	χ2=21.6 *p*<0.001
Bothered a little	35 (33.7)	69 (66.3)	104 (37.3)	
Bothered a lot	6 (9.2)	59 (90.8)	65 (23.3)	
***Pain or problems during sexual intercourse***				
Not bothered at all	54 (37.8)	89 (62.2)	143 (51.2)	χ2=11.3 *p*=0.004
Bothered a little	27 (32.9)	55 (67.1)	82 (29.4)	
Bothered a lot	7 (13.0)	47 (87.0)	54 (19.4)	
***Constipation, loose bowels, or diarrhea***				
Not bothered at all	47 (37.9)	77 (62.1)	124 (44.4)	χ2=7.92 *p*=0.019
Bothered a little	33 (31.4)	72 (68.6)	105 (37.6)	
Bothered a lot	8 (16.0)	42 (84.0)	50 (18.0)	
***Nausea, gas, or indigestion***				
Not bothered at all	42 (32.6)	87 (67.4)	129 (46.2)	χ2=4.07 *p*=0.131
Bothered a little	36 (36.0)	64 (64.0)	100 (35.8)	
Bothered a lot	10 (20.0)	40 (80.0)	50 (18.0)	

Characteristics	Non-case of PTSD n (%)	A case of PTSD n (%)	Total n (%)	χ2 *P*-value
***Feeling tired or having low energy***				
Not bothered at all	29 (41.4)	41 (58.6)	70 (25.1)	χ2=31.7 *p*<0.001
Bothered a little	50 (43.5)	65 (56.5)	115 (41.2)	
Bothered a lot	9 (9.6)	85 (90.4)	94 (33.7)	
***Trouble sleeping***				
Not bothered at all	37 (46.8	42 (53.2)	79 (28.3)	χ2=37.1 *p*<0.001
Bothered a little	43 (41.3)	61 (83.9)	104 (37.3)	
Bothered a lot	8 (08.3)	88 (91.7)	96 (34.4)	

Values are presented as number and percentage (%). *Mean score >2.5 on the Harvard Trauma Questionnaire (HTQ)-16’s sub-items or entire scale (HTQ-45) indicated a case of PTSD, χ2: Chi-square, *p*: significant level

**Figure 1:**
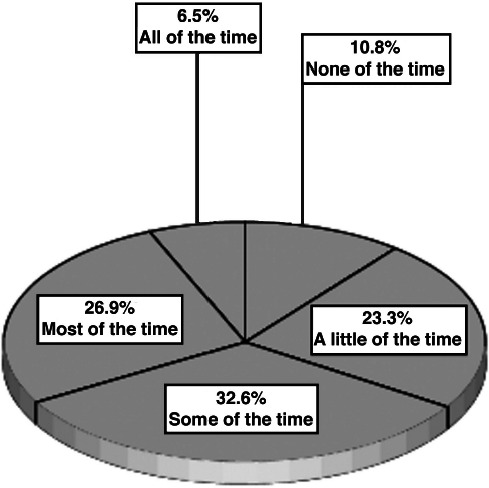
- The effect of physical symptoms or psychological problems on social activities among participants during the last 4 weeks’ time frame.

Figure 1 illustrates how physical or psychological symptoms of PTSD, if present, affect the social activities of the participants during the last 4 weeks. Almost 27% of the participants reported that these symptoms affected their social activities most of the time, and 7% reported that their physical and psychological symptoms affected their social activities all the time.

According to the results of a multivariate-adjusted logistic regression, a test of the whole model including all 21 related factors was statistically significant and fit data well, when compared to the null model, with a value of χ^2^(22)=137.15, *p*<0.001 and a Pearson Chi-square statistic for goodness-of-fit test of χ^2^(242)=231.5, *p*=0.676. For each of the associated variables, Table 4 presents the anticipated logistic regression coefficients, Wald statistics, odds ratio of being a case of PTSD, as well as a 95% CI. The entire model explained between 39.2% and 55% of the variance that can be predicted from the independent set of variables, according to the results of the Cox & Snell and Nagelkerke R^2^ estimations. With an overall classification success rate of 85.1%, the model correctly identified 92.6% of participants who were a case of PTSD and 69.3% were not.

**Table 4:** - Predicting the likelihood of being of a case of PTSD using a multivariate logistic regression* (n= 279).

Predictor variables	B	Wald	p	OR	95% CI
***Age***					
Young (18-30)	0.993	2.51	0.130	2.70	0.746 –9.76
Middle-aged (31- 45)	1.87	7.31	0.007	6.89	1.62 –26.1
Old-aged (>45)	Ref.				
***Gender***					
Female	-1.43	9.94	0.002	0.239	0.098–0.582
Male	Ref				
***Employment status***					
Unemployed	-0.876	4.54	0.033	0.416	0.186–0.932
Employed	Ref.				
***A family member died during the conflict***					
No	1.74	17.0	0.000	5.7	2.49–13.0
Yes	Ref.				
***If peace restore you will go back home***					
No	-0.234	0.194	0.660	0.792	0.279–2.24
Yes	Ref.				
***“Witnessed mass execution for civilians”***					
No	0.530	1.15	0.285	1.7	0.644–4.48
Yes	Ref.				
***“Witnessed shelling, burning, or razing of residential areas or marshlands”***					
No	1.96	12.1	0.001	7.12	2.35–21.6
Yes	Ref.				
***“Witnessed chemical attacks on residential areas or marshlands”***					
No	0.747	1.69	0.193	2.11	0.685 – 6.51
Yes	Ref.				
***“Serious physical injury of family member or friend from combat situation or landmine”***					
No	0.358	0.694	0.405	1.43	0.616 – 3.32
Yes	Ref.				
***“Witnessed rotting corpses”***					
No	0.182	0.136	0.712	1.20	0.457–3.15
Yes	Ref.				
***“Witnessed sexual abuse or rape”***					
No	1.04	3.08	0.079	2.82	0.886–9.00
Yes	Ref.				

Predictor variables	B	Wald	p	OR	95% CI
***“Witnessed torture”***					
No	0.472	1.12	0.289	1.60	0.670–3.84
Yes	Ref.				
***“Witnessed murder”***					
No	-0.123	0.062	0.804	0.884	0.335–2.34
Yes	Ref.				
***“Murder or violent death of a family member”***					
No	-.870	3.26	0.071	0.419	0.16 –1.08
Yes	Ref.				
***“Received the body of a family member and prohibited from mourning or performing burial rites”***					
No	1.32	5.80	0.016	3.73	1.28 –10.88
Yes	Ref.				
***“Disappearance of a family member or a friend”***					
No	.895	3.62	0.057	2.45	0.973 – 6.16
Yes	Ref.				
***“Family member or friend kidnapped”***					
No	-.557	1.20	0.273	0.573	0.212 – 1.55
Yes	Ref.				
***“Family member or friend taken as a hostage”***					
No	0.479	0.864	0.354	1.61	0.588 – 4.43
Yes	Ref.				
“Kidnapped”					
No	-2.24	3.61	0.058	0.106	0.011 – 1.07
Yes	Ref.				
***“Sexually abused or raped (such as, forced sexual activity)”***					
No	2.05	7.57	0.006	7.75	1.78 – 33.4
Yes	Ref.				
***“Tortured”***					
No	0.442	0.447	0.504	1.56	0.426 – 5.69
Yes	Ref				

*Mean scores >2.5 in the sub-items (Harvard Trauma Questionnaire [HTQ]-16) or the entire scale HTQ-45 indicated a case of PTSD, B: estimated multinomial logistic regression coefficients, *p*: significant level, OR: Odds ration, CI: confidence interval Ref.: reference group

As showed in Table 4, only 7 associated variables, out of the 21 variables, showed a significant relationship with being a case of PTSD based on the Chi-square values presented in Tables 1 & 2, made unique statistically significant contributions for the likelihood of being a case of PTSD, while holding all other variables in the model constant. The odds ratios are presented in Table 4 (column 4), displayed the likelihood of being a case of PTSD. The factor with the strongest correlation of being a case of PTSD was “sexually abused or raped (such as forced sexual activity)”. It recognized an odds ratio equal to 7.75 indicating that a participant who experienced being sexually abused or raped is almost 8 times more likely to be considered a case of PTSD than a participant who had not, when all the other predictors in the model were held constant. The variable “witnessed shelling, burning, or razing of residential areas or marshlands” showed odds ratio for being a case of PTSD of 7.12, and specifies that participants witnessing such trauma are approximately 7 times more likely to be a case of PTSD compared to those who had not witnessed such events. Among age groups, participants who were middle-aged (3-45 years old) presented odds ratio of 6.89 indicating that those who were middle- aged were approximately 7 times more likely to be considered a case of PTSD than those who were older in age, holding constant all other variables in the model.

The independent variables of losing a family member during the conflict, and “receiving the body of a family member and prohibited from mourning or performing burial rites” showed an odds ratios of 5.7, and 3.73, indicating that those who went through this experience were approximately 6 times and 4 times, more likely to be considered a case of PTSD than those who did not (while all other variables in the model held constant).

The Wald ratio for the coefficient related to being a female was statistically significant, B= -1.43, Wald χ^2^(1)=9.94, *p*=0.002, indicating a significant difference in odd of being a case of PTSD among female participants compared to male. The female odds ratio was 0.239 specifying that when all related variables were held constant, females were less likely to be classified as a non-case of PTSD than males. Also, the Wald ratio for the coefficient associated with being unemployed was statistically significant, B= -876, Wald χ^2^(1)=4.54, *p*=0.033, revealing that there was a significant difference in odds of being considered a case of PTSD among unemployed participants compared with employed ones. The odds ratio for unemployed was 0.416, indicating that being unemployed was less likely to be considered a non-case of PTSD than those who were employed (keeping constant all associated variables).

After settling in Jordan, approximately 63% of the participants were not satisfied with their income. Almost 32% of the participants were not satisfied with the medical care provided to them and rated it as bad. Hence, 40% of them reported that the psychological care provided was poor. Further, 41% of participants reported that their freedom to practice their religion was excellent. Table 5 shows the level of satisfaction reported by the participants regarding the different services provided during their presence in Jordan.

**Table 5:** - The study participants’ level of satisfaction with the services provided to them during being settled in Jordan (N=279).

Type of service	Bad n (%)	Accepted n (%)	Medium n (%)	Good n (%)	Excellent n (%)
Food	56 (20.1)	106 (38.0)	62 (22.2)	39 (14.0)	16 (5.7)
Housing	88 (31.5)	102 (36.5)	51 (18.3)	32 (11.5)	6 (2.2)
Income	177 (63.4)	56 (20.1)	34 (12.2)	11 (3.9)	1 (0.4)
Environment around in general	66 (23.7)	99 (35.5)	70 (25.1)	37 (13.3)	7 (2.5)
Freedom of religious practice	9 (3.2)	39 (14.0)	31 (11.1)	87 (31.2)	113 (40.5)
Medical care	91 (32.6)	88 (31.5)	56 (20.1)	38 (13.6)	6 (2.2)
Psychological care	112 (40.1)	86 (30.8)	54 (19.4)	25 (9.0)	2 (0.7)
Care of medicine or drugs	97 (34.8)	80 (28.7)	54 (19.4)	45 (16.1)	3 (1.1)
Children general care	67 (24.0)	79 (28.3)	64 (22.9)	51 (18.3)	18 (6.5)
Children teaching	90 (32.3)	59 (21.1)	63 (22.6)	52 (18.6)	15 (5.4)
Children healthcare	65 (23.3)	75 (26.9)	63 (22.6)	60 (21.5)	16 (5.7)
Children feeding	72 (25.8)	73 (26.2)	60 (21.5)	53 (19.0)	21 (7.5)
Provide suitable activities to children	58 (20.8)	74 (26.5)	54 (19.4)	50 (17.9)	43 (15.4)

## Discussion

Refugees are likely to face various physical and psychological traumatic events that might lead to PTSD such as and not limited to sexual abuse or rape, and witnessing destruction or the killing of innocent people. This study demonstrated the prevalence, possible stressors, physical and psychological symptoms associated with PTSD, and quality of life reported by Syrian refugees residing in non-camp settings in Jordan. This study reported high rates of traumatic symptoms among the Syrian refugees (86.2%), of whom many (68.5%) were experiencing symptoms to be considered as a case of PTSD. Consistent with our study results, other studies have reported higher PTSD prevalence among Syrian refugees in times of conflict.^1,26^ Indeed, PTSD was among the health related concerns reported by Syrian refugees who resided in non-camp setting.^17^

The high prevalence of PTSD among the study participants could be related to their age. The majority of the study participants were middle-aged, with a mean age of 32 years. In comparison to older participants, the middle-aged participants were more likely to report symptoms to be considered as a case of PTSD. Along with depression, PTSD has been reported to be a rising problem among young adult Syrian refugees resided in non-camp settings in Jordan.^17^ War is well known to create various traumatic experiences, such as war trauma and torture, which can lead to the development of PTSD.^27^ Several studies have reported that people in war-affected zones experience at least one traumatic event due to political conflict and war, and several traumatic events have been associated with PTSD, including home confinement due to chaos and violence, death of a family member, and catastrophic experiences of being kidnapped, sexually abused, or raped.^27,28^ Participants in the current study reported 5014 different types of traumas, and over half of them reported a minimum of one family member’s death during the war. Reporting sexual abuse or rape was the strongest predictor of being a case of PTSD in the current study, in addition to other types of trauma like losing a family member in the conflict, witnessing catastrophic events like burning, or razing of residential areas, and receiving the body of a family member while being prohibited from expressing grief and performing funeral rites. This finding is supported by previous findings which reported sexual abuse and violence to be the most potentially traumatizing experiences with the highest post-traumatic risk for developing PTSD, especially among women.^29,30^ According to a study carried out among Syrian Kurdish refugees in Iraq’s Kurdistan region, home confinement due to disorder and violence was one of the most common traumatic incidents reported by refugees (62%).^31^ In addition, forced to flee their country (87%) and “witnessing shelling, burning, or razing of residential areas or marshlands” (65%) were also reported among the most frequent traumatic events experienced.^31^ Other studies reported other risk factors of developing PTSD, like migration trauma, sexual violence, child-rearing pressures, and safety concerns, particularly among women.^26^

Female participants and those who were unemployed were found to be more likely to be considered as a case of PTSD. An earlier study assessed mental disorders among refugees reported that refugees rejected for employment (such as unemployed refugees) were 1.35 times more likely to develop PTSD for each additional rejection and twice likely to have a major depressive disorder.^32^ Employment has been reported to reduce stress and anxiety by increasing the sense of well and control and enhancing financial capability, which helps improve a person’s psychological symptoms.^10,14,33^ The current study also supports previous study findings that demonstrated a high correlation between the occurrence of mental health problems like PTSD and sexual abuse and unemployment among female rape survivors.^15,34^

Interestingly, the results of this study indicate that almost 80% of refugees’ were willing to return to their country after the war ended and this was significantly correlated with being a case of PTSD. According to literature, this outcome was expected; traumatic stresses encountered during wars, vulnerability, harsh living conditions, and the inability to cope with new stressors impact refugees’ mental well-being and, as a result, their desire to return to their country of origin. Similar findings were reported among refugees in Germany, where considerable correlations were found between refugees’ living situations, desire to return, and mental well-being.^35^ However, according to this study the return decision for two-thirds of refugees was not voluntary.^35^

Several physical or psychological symptoms of PTSD have been reported among refugees, affecting their social activities and quality of life. Such physical symptoms included headaches, pain in their arms, legs, or joints, feeling tired, or having low energy and sleep disturbances, in addition to menstrual cramps or other period-related problems in women. A previous study of Syrian refugees in Jordan has also reported up to 40% of participants were newly diagnosed with chronic diseases accompanied by a risk of mental problems like depression, which was found to be associated with PTSD.^34,36^ The connection between psychological, physical, and social well-being has been reported previously. Social health among displaced Syrians and Jordanians was positively correlated with better physical health and negatively correlated with PTSD symptoms, highlighting the interconnection between psychological and physical health.^37^

Outcomes indicating refugee’s quality of life showed that 32% were not satisfied with the medical care provided to them, and 40% reported that the psychological care provided to them was suboptimal. This could be related to the fact that a large number of Syrian refugees have been displaced to Jordan, which has severely affected the health care system and the health infrastructure, along with the high utilization of health care services of the Syrian refugees. Thus, the burden on the healthcare system in Jordan is very high.^17,18^ However, Syrian refugees, particularly those residing in non-camp settings, could experience poor health associated with mental health problems like PTSD, and this could be undertreated due to troubles in obtaining medical care as a result of their situations and lack of health insurance, the cessation of free access to health services, healthcare costs as well as cultural differences.^10,19,20^ Similar to our findings, it has been reported that increased attention given to the healthcare sector to provide more support to Syrian refugees in Sweden and other European countries provided better access and continuity to the healthcare services.^16,38,39^ Our study sheds light on those who are in urgent need of assistance, mainly who have symptoms that predict PTSD, in addition to highlighting the importance of providing medical centers and access to these centers by refugees in need of such care.

### Study limitations

This study is limited to its cross-sectional design used to evaluate PTSD symptoms in a convenient sample at a single point of time, as it is difficult to evaluate how PTSD symptoms change with time. Therefore, the generalizability of the study findings may be limited. However, the study sample was quite large and diverse as it was gathered from several Jordanian cities and towns and included only one participant from each family. In this study, PTSD was evaluated using a self-reported scale, which exposes patients to the possibility of exaggeration and the possibility of embarrassment, which may prevent them from disclosing intimate aspects of their experience, providing socially acceptable responses. However, limited studies were carried out in Jordan, its’ findings can serve as a baseline for subsequent studies into this subject. Except for having a medical history of psychiatric and mental disorders, which were used as exclusion criteria in the current study, the medical history of other illness among the study participants, prior to their residence in Jordan, were not assessed. Since several medical conditions may affect the physical symptoms of PTSD that were measured in this study, future studies assess other physical symptoms of PTSD that were not covered in the current study, as well as to assess the effects of other medical conditions among the Syrian refugees, is recommended.

In conclusion, majority of the refugees residing in Jordan suffer from PTSD. The most common type of trauma reported was expulsion from their country based on ancestral origin, religion, or sector. This study identified several factors associated with PTSD among Syrian refugees residing in non-camp settings in Jordan; those who were females, middle-aged, unemployed, sexually abused or raped, witnessed catastrophic events like burning, or razing of residential areas, had a family member who died in the conflict, received the body of a family member while being prohibited from expressing grief and doing funeral rites, were more likely to report symptoms to be considered as a case of PTSD. The study reported important psychological, physical, and social impacts on Syrian refugees’ daily activities and quality of life. Majority of participants reported experiencing some level of a bothered headache and a lot of pain in their arms, legs, or joints. Refugees showed sub satisfaction with their income and healthcare services provided, including psychological care. The findings of this study highlight the profound need to implement new policies and interventions supporting refugees’ mental health, particularly among young adults, women, unemployed and those who have experienced severe or multiple traumatic events.

## References

[1] Basheti IA , Ayasrah SM , Basheti MM , Mahfuz J , Chaar B. The Syrian refugee crisis in Jordan: a cross sectional pharmacist-led study assessing post-traumatic stress disorder. Pharm Pract (Granada) 2019; 17: 1475. 31592018 10.18549/PharmPract.2019.3.1475 PMC6763294

[2] Global Trends forced displacement in 2017. (Updated: 2017; accessed 2019 Nov 11). Available from: https://www.unhcr.org/5b27be547.pdf

[3] United Nations population Fund regional situation report for the Syrian crisis. (Updated 2019; accessed 2019 Nov 11). Available from: https://data2.unhcr.org/en/situations/syria/location/36

[4] Sundquist J , Behmen-Vincevic A , Johansson SE. Poor quality of life and health in young to middle aged Bosnian female war refugees: a population-based study. Public Health 1998; 112: 21-26. 9490884 10.1038/sj.ph.1900411

[5] Akinyemi OO , Owoaje ET , Ige OK , Popoola OA. Comparative study of mental health and quality of life in long-term refugees and host populations in Oru-Ijebu, Southwest Nigeria. BMC Res Notes 2012; 5: 394. 22846111 10.1186/1756-0500-5-394 PMC3461488

[6] Eisenman DP , Gelberg L , Liu H , Shapiro MF. Mental health and health-related quality of life among adult Latino primary care patients living in the United States with previous exposure to political violence. JAMA 2003; 290: 627-634. 12902366 10.1001/jama.290.5.627

[7] Freitag S , Braehler E , Schmidt S , Glaesmer H. The impact of forced displacement in World War II on mental health disorders and health-related quality of life in late life - a German population-based study. Int Psychogeriatr 2013; 25: 310-319. 22999056 10.1017/S1041610212001585

[8] Ikin JF , Sim MR , McKenzie DP , Horsley KW , Wilson EJ , Harrex WK , et al. Life satisfaction and quality in Korean War veterans five decades after the war. J Epidemiol Community Health 2009; 63: 359-365. 19366889 10.1136/jech.2007.061986

[9] Laban CJ , Komproe IH , Gernaat HB , de Jong JT. The impact of a long asylum procedure on quality of life, disability and physical health in Iraqi asylum seekers in the Netherlands. Soc Psychiatry Psychiatr Epidemiol 2008; 43: 507-515. 18560785 10.1007/s00127-008-0333-1

[10] Aziz IA , Hutchinson CV , Maltby J. Quality of life of Syrian refugees living in camps in the Kurdistan region of Iraq. Peer J 2014; 2: e670. 25401057 10.7717/peerj.670 PMC4230548

[11] Bogic M , Njoku A , Priebe S. Long-term mental health of war-refugees: a systematic literature review. BMC Int Health Hum Rights 2015; 15: 29. 26510473 10.1186/s12914-015-0064-9 PMC4624599

[12] Fazel M , Wheeler J , Danesh J. Prevalence of serious mental disorder in 7000 refugees resettled in western countries: a systematic review. Lancet 2005; 365: 1309-1314. 15823380 10.1016/S0140-6736(05)61027-6

[13] Norredam M , Garcia-Lopez A , Keiding N , Krasnik A. Risk of mental disorders in refugees and native Danes: a register-based retrospective cohort study. Soc Psychiatry Psychiatr Epidemiol 2009; 44: 1023-1029. 19294322 10.1007/s00127-009-0024-6

[14] Tol WA , Kohrt BA , Jordans MJ , Thapa SB , Pettigrew J , Upadhaya N , et al. Political violence and mental health: a multi-disciplinary review of the literature on Nepal. Soc Sci Med 2010; 70: 35-44. 19833427 10.1016/j.socscimed.2009.09.037

[15] Matanov A , Giacco D , Bogic M , Ajdukovic D , Franciskovic T , Galeazzi GM , et al. Subjective quality of life in war-affected populations. BMC Public Health 2013; 13: 624. 23819629 10.1186/1471-2458-13-624 PMC3716711

[16] Leiler A , Bjarta A , Ekdahl J , Wasteson E. Mental health and quality of life among asylum seekers and refugees living in refugee housing facilities in Sweden. Soc Psychiatry Psychiatr Epidemiol 2019; 54: 543-551. 30580381 10.1007/s00127-018-1651-6

[17] Al-Rousan T , Schwabkey Z , Jirmanus L , Nelson BD. Health needs and priorities of Syrian refugees in camps and urban settings in Jordan: perspectives of refugees and health care providers. East Mediterr Health J 2018; 24: 243-253. 29908019 10.26719/2018.24.3.243

[18] Doocy S , Lyles E , Akhu-Zaheya L , Burton A , Burnham G. Health service access and utilization among Syrian refugees in Jordan. Int J Equity Health 2016; 15: 108. 27418336 10.1186/s12939-016-0399-4 PMC4946096

[19] Dator W , Abunab H , Dao-Ayen N. Health challenges and access to health care among Syrian refugees in Jordan: a review. East Mediterr Health J 2018; 24: 680-686. 30215478 10.26719/2018.24.7.680

[20] Ay M , Arcos González P , Castro Delgado R. The Perceived Barriers of Access to Health Care Among a Group of Non-camp Syrian Refugees in Jordan. Int J Health Serv 2016; 46: 566-589. 26962004 10.1177/0020731416636831

[21] Kleijn WC , Hovens JE , Rodenburg JJ. Posttraumatic stress symptoms in refugees: assessments with the Harvard Trauma Questionnaire and the Hopkins symptom Checklist-25 in different languages. Psychol Rep 2001; 88: 527-532. 11351903 10.2466/pr0.2001.88.2.527

[22] Mollica RF , Harvard Program in Refugee T. Measuring trauma, measuring torture : instructions and guidance on the utilization of the Harvard Program in Refugee Trauma’s versions of The Hopkins Symptom Checklist-25 (HSCL-25) & the Harvard Trauma Questionnaire (HTQ). Cambridge, MA: Harvard Program in Refugee Trauma; 2004.

[23] Shoeb M , Weinstein H , Mollica R. The Harvard trauma questionnaire: adapting a cross-cultural instrument for measuring torture, trauma and posttraumatic stress disorder in Iraqi refugees. Int J Soc Psychiatry 2007; 53: 447-463. 18018666 10.1177/0020764007078362

[24] Mollica RF , McDonald LS , Massagli MP , Silove DM. Measuring Trauma , Measuring Torture: Instructions and Guidance on the Utilization of the Harvard Program in Refugee Trauma’s Versions of The Hopkins Symptom Checklist-25 (HSCL-25) & The Harvard Trauma Questionnaire (HTQ). Cambridge, (MA); 2004.

[25] McFarlane AC , Atchison M , Rafalowicz E , Papay P. Physical symptoms in post-traumatic stress disorder. J Psychosom Res 1994; 38: 715-726. 7877126 10.1016/0022-3999(94)90024-8

[26] Ditlevsen DN , Elklit AJAogp. Gender, trauma type, and PTSD prevalence: a re-analysis of 18 nordic convenience samples. Ann Gen Psychiatry 2012; 11: 26. 23107002 10.1186/1744-859X-11-26 PMC3494556

[27] Johnson H , Thompson AJCpr. The development and maintenance of post-traumatic stress disorder (PTSD) in civilian adult survivors of war trauma and torture: A review. Clin Psychol Rev 2008; 28: 36-47. 17383783 10.1016/j.cpr.2007.01.017

[28] Mahmood HN , Ibrahim H , Goessmann K , Ismail AA , Neuner FJC , health. Post-traumatic stress disorder and depression among Syrian refugees residing in the Kurdistan region of Iraq. Conflict and Health 2019; 13: 1-11. 31728157 10.1186/s13031-019-0238-5 PMC6842196

[29] Tolin DF , Foa EB. Sex differences in trauma and posttraumatic stress disorder: a quantitative review of 25 years of research. Psychol Bull 2006; 132; 959-992. 10.1037/0033-2909.132.6.959 17073529

[30] Irish LA , Fischer B , Fallon W , Spoonster E , Sledjeski EM , Delahanty DLJJoAD. Gender differences in PTSD symptoms: an exploration of peritraumatic mechanisms. J Anxiety Disord 2011; 25: 209-216. 20956066 10.1016/j.janxdis.2010.09.004 PMC3031659

[31] Ibrahim H , Hassan CQ. Post-traumatic Stress Disorder symptoms resulting from torture and Other traumatic events among Syrian Kurdish refugees in Kurdistan region, Iraq. Front Psychol 2017; 8: 241. 28265252 10.3389/fpsyg.2017.00241 PMC5316552

[32] Hocking DC , Kennedy GA , Sundram S. Mental disorders in asylum seekers: The role of the refugee determination process and employment. J Nerv Ment Dis 2015; 203: 28-32. 25503784 10.1097/NMD.0000000000000230

[33] Paul K , Moser K. Unemployment impairs mental health: meta-analyses. J Organ Behav 2009; 74: 264-282.

[34] Mgoqi-Mbalo N , Zhang M , Ntuli S. Risk factors for PTSD and depression in female survivors of rape. Psychol Trauma 2017; 9: 301-308. 28114775 10.1037/tra0000228 PMC5411289

[35] von Lersner U , Elbert T , Neuner F. Mental health of refugees following state-sponsored repatriation from Germany. BMC Psychiatry 2008; 8: 88. 19000300 10.1186/1471-244X-8-88 PMC2596775

[36] Gammouh OS , Al-Smadi AM , Tawalbeh LI , Khoury LS. Chronic diseases, lack of medications, and depression among Syrian refugees in Jordan, 2013-2014. Preventing chronic disease 2015; 12: E10. 25633485 10.5888/pcd12.140424 PMC4310712

[37] Powell TM , Shin OJ , Li S-J , Hsiao Y. Post-traumatic stress, social, and physical health: A mediation and moderation analysis of Syrian refugees and Jordanians in a border community. PLOS ONE 2020; 15: e0241036. 33095832 10.1371/journal.pone.0241036 PMC7584168

[38] van Loenen T , van den Muijsenbergh M , Hofmeester M , Dowrick C , van Ginneken N , Mechili EA , et al. Primary care for refugees and newly arrived migrants in Europe: a qualitative study on health needs, barriers and wishes. Eur J Public Health 2018; 28: 82-87. 29240907 10.1093/eurpub/ckx210

[39] Tinghög P , Malm A , Arwidson C , Sigvardsdotter E , Lundin A , Saboonchi FJBo. Prevalence of mental ill health, traumas and postmigration stress among refugees from Syria resettled in Sweden after 2011: a population-based survey. BMJ Open 2017; 12: e018899. 10.1136/bmjopen-2017-018899 PMC5778338 29289940

